# Heavy Metals in Foods Consumed by Copper Miners: A Health Risk Assessment

**DOI:** 10.1002/fsn3.70267

**Published:** 2025-05-09

**Authors:** Maryam Rostamzadeh, Elham Khalili Sadrabad, Fateme Akrami Mohajeri, Arefeh Dehghani‐tafti, Elaheh Askari

**Affiliations:** ^1^ Department of Food Hygiene and Safety, School of Public Health Shahid Sadoughi University of Medical Sciences Yazd Iran; ^2^ Research Center for Food Hygiene and Safety, Department of Food Science and Technology, School of Public Health Shahid Sadoughi University of Medical Sciences Yazd Iran; ^3^ Department of Biostatistics, School of Public Health Shahid Sadoughi University of Medical Sciences Yazd Iran; ^4^ Nutritional Health Research Center, School of Health and Nutrition Lorestan University of Medical Sciences Khorramabad Iran

**Keywords:** food safety, heavy metals, miners, occupational exposure, risk assessment

## Abstract

Due to the high occupational exposure of miners to heavy metals, the concentration of arsenic, cadmium, lead, copper, and zinc in raw and cooked foods consumed by mine workers and the risk assessment of the consumption of these foods were investigated. A total of 105 samples of raw and cooked foods and beverages were collected. Samples were oven‐dried, digested with microwave‐assisted nitric acid for the analysis of heavy metals using an ICP‐OES. The health risk assessment was performed through hazard quotient (HQ) and hazard index (HI) calculations. The concentrations of all heavy metals measured in both raw foods and cooked foods were lower than the permissible limits as determined by FAO/WHO and Iranian national standards. The HQ and HI for all metals were less than one, signifying no health risk from dietary exposure. Heavy metal levels in food taken by miners fell within permissible limits, but periodic monitoring is necessary because of miners' occupational exposure to heavy metals.

## Graphical abstract

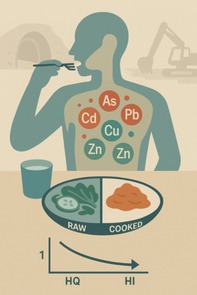

## 1 Introduction

The industrial revolution and ongoing human activities have significantly impacted our environment, leading to widespread contamination from heavy metals. These pollutants pose serious risks to both ecosystems and human health, particularly as they can enter the food chain. In recent years, increasing concerns have emerged regarding how heavy metals infiltrate agricultural systems, ultimately affecting food safety (Zaynab et al. 2022). Heavy metals, such as cadmium (Cd), lead (Pb), mercury (Hg), and arsenic (As), are of particular concern due to their toxicity and persistence in the environment. For instance, studies have shown that agricultural soils in mining regions often exhibit elevated levels of these metals because of mining activities and the use of contaminated fertilizers (Zazouli et al. 2008). Furthermore, groundwater can become contaminated with heavy metals as a result of industrial activities (Ghazanfarirad et al. 2014).

Research in various mining areas has documented significant health risks associated with heavy metal exposure, highlighting the urgent need for comprehensive assessments. For example, in regions like the Zambian Copper belt, studies have reported alarming levels of lead and cadmium in both soil and crops, leading to serious public health implications (Dusengemungu et al. 2022). Similarly, research in the Andes has demonstrated that communities living near mining operations are at increased risk of heavy metal contamination in their diets, resulting in various health issues (Custodio et al. 2020). In addition, the improper food preparation, processing, and storage, along with the lack of proper health management, have led to an increase in the possibility of food contamination by heavy metals (Ebrahimi et al. 2020). Heavy metals are defined as nonbiodegradable, nonessential elements and toxic contaminants. It is well documented that they have the capacity to enter the human body via food intake (Senila 2023). Heavy metals, including Pb, Cd, and mercury (Hg) and arsenic (As), have been associated with immune deficiency, cardiovascular problems, neurotoxicity, carcinogenicity, and teratogenicity effects (Fang et al. 2014). For the sake of simplicity, As was included in the list of heavy metals, despite its classification as a metalloid (Senila 2023).

Health risk assessment (HRA) is an essential tool used to evaluate the potential health impacts of hazardous substances, including heavy metals. HRA involves a systematic process that includes hazard identification, dose–response assessment, exposure assessment, and risk characterization. According to the World Health Organization (2021), HRA provides a comprehensive understanding of the potential dangers faced by at‐risk populations.

The workers, especially the mining workers, are in direct contact with heavy metals for a long time through inhalation and skin contact (Fatima et al. 2024). There is not much information about the heavy metals in the food served to mining workers. Therefore, due to the high occupational exposure of mine workers to heavy metals, it is very important to ensure the health and safety of food consumed by this group. As the Copper industry is one of the largest industries in Iran, the aim of the current study was to investigate the heavy metals concentration in food consumed (usual meals) by the mining workers. In this study, we will apply HRA methodologies to assess the levels of heavy metals in food consumed by mining workers and quantify the associated health risks. By systematically identifying and analyzing the concentrations of these contaminants in their food, this research aimed to illuminate the health implications for mining communities. Ultimately, the findings will contribute to public health strategies aimed at minimizing exposure and safeguarding the health of workers in the copper mining sector.

## 2 Material and Method

### 2.1 Sample Preparation

In this study, raw foodstuffs including rice, veal, lamb meat, turkey, fish, shrimp, chicken, spices, cobs, vegetables, lentils, green beans, eggplant, tomato paste, pinto beans, and oil, were randomly sampled. The cooked food samples, including bread, turkey, lamb, rice + lentil + meat, Istanbuli polo (rice with meat and bean), minced kebab, Qormeh‐Sabzi stew, Gheimeh stew (split peas), Eggplant stew, chicken, chicken barbecue, fried fish, fried shrimp, and cooked rice, were collected. Drinking water, juice, doogh, soft drink, and soda were also sampled. The samples were collected in a PET (polyethylene terephthalate) container. They were kept away from light and taken to the laboratory.

### 2.2 Heavy Metal Analysis

#### 2.2.1 Reagent

All used chemical reagents were of analytical grade. The glasswares were soaked in diluted nitric acid (10%) overnight and then rinsed with distilled deionized water.

#### 2.2.2 Microwave Digestion

Food samples were dried in an oven at 60°C for 24 h. A total of 0.1 g of homogeneous powdered samples was transferred to the vessel, and 5 mL of HNO_3_ (65%) was added. Samples were digested by a microwave digestion system (Anton Paar, Austria) for 20 min (temperature of 180°C and power of 900 W). The digested samples were diluted to 10 mL with distilled deionized water and filtered through a 0.45 μ filter membrane.

#### 2.2.3 Heavy Metal Analysis

The heavy metal concentrations were determined by Inductively Coupled Plasma‐Optical Emission Spectrophotometry (ICP‐OES, Agilent 5110, USA). Certified standard solutions from Merck were used for calibration ICP‐OES. These solutions included the target elements at a concentration of 1000 mg/L in 2% nitric acid. The standard solutions for the calibration curve were prepared by appropriate dilution of the stock solutions with ultrapure deionized water (18.2 MΩ·cm) to obtain standard concentrations of 0.1, 0.5, 1, 5, and 10 mg/L; fresh calibration standards were prepared daily prior to analysis. The blank solution (2% HNO_3_) was also applied to set the baseline. All solutions were kept in acid‐washed polypropylene containers to avoid cross‐contamination. These measurements were compared with a known reference generated by quality control standards in order to validate calibration.

### 2.3 Health Risk Assessment

To determine the health risk assessment, the Estimated Daily Intake (Famurewa et al. 2023) of Metals was calculated according to the following equation:

(1) $$\begin{array}{cc} \mathrm{EDI}=\; & \text{daily food consumption}\;\left(g\right)\times \text{concentration}\;\\ & \text{of metal}\left(\mathrm{mg}\;{\mathrm{kg}}^{-1}\right)/\text{body weight} \end{array}$$

Then, the hazard quotient (HQ) for noncarcinogenic risk of metal was evaluated as the following equation:

(2) HQ=EDI/RfD

According to FAO/WHO, the oral toxicity reference dose values (RfD) are 0.3, 1, 40, 300, and 3.75 μg/kg for As, Cd, Cu, Zn, and Pb, respectively.

Hazard index (HI): The HI, which shows the total noncarcinogenic health risk of heavy metals, was calculated as below (Pourramezani et al. 2019):

(3) $$\mathrm{HI}={\mathrm{HQ}}_{1}+{\mathrm{HQ}}_{2}+{\mathrm{HQ}}_{3}+\ldots +{\mathrm{HQ}}_{n}$$

### 2.4 Statistical Analysis

The data were analyzed by SPSS V21 software. The results were analyzed using descriptive statistics (mean and standard deviation) and analytical statistics (ANOVA and *t*‐test) at a 95% confidence level.

## 3 Results and Discussion

Soil in regions where mining activities are conducted typically contains high levels of heavy metals. Previous studies have indicated that mine workers are particularly susceptible to heavy metal toxicity (Mousavian et al. 2017). Therefore, in the present study, the concentration of heavy metals Cu, As, Zn, Pb, and Cd in raw and cooked food and beverages consumed by mine workers of copper mines was determined.

### 3.1 Heavy Metals in Raw Foodstuffs

The concentration of As in the samples varied from the lowest value in the samples of split peas and lentils to the highest value in the samples of veal and lamb meat (Table 1). The higher Cd concentration in the samples was in pinto beans and tomato paste. The Pb in the lamb meat and tomato paste samples was higher. The lowest and highest concentration of Cu was estimated in oil and shrimp, respectively. Similarly, the lowest and highest levels of Zn were in the oil and veal meat, respectively.

**TABLE 1:** Concentration of heavy metals in raw food based on dry weight (mean ± SD).

Foodstuffs	As (μg/kg)	Cd (μg/kg)	Pb (μg/kg)	Cu (mg/kg)	Zn (mg/kg)
Rice	0.044 ± 0.002^d^	0.044 ± 0.001^a^	0.044 ± 0.001^c^	8.96 ± 0.67^f^	2.42 ± 4.48^k^
Green beans	3.76 ± 0.10^c^	0.037 ± 0.001^c^	3.75 ± 0.12^a^	18.78 ± 2.21^c^	26.29 ± 0.01^f^
Lentil	0.030 ± 0.002^e^	0.030 ± 0.001^d^	0.030 ± 0.001^d^	12.30 ± 3.07^e^	39.97 ± 3.07^e^
Pinto beans	0.041 ± 0.002^d^	0.41 ± 0.003^a^	0.041 ± 0.003^c^	12.15 ± 1.23^e^	16.57 ± 4.14^h^
Split peas	0.030 ± 0.002^e^	0.030 ± 0.001^d^	0.030 ± 0.001^d^	12.01 ± 1.27^e^	33.54 ± 4.00^e^
Veal	6.31 ± 0.24^a^	0.042 ± 0.003^b^	0.042 ± 0.002^c^	16.95 ± 0.20^d^	143.82 ± 4.23^a^
Lamb meat	4.50 ± 0.18^b^	0.045 ± 0.002^b^	4.5 ± 0.94^a^	13.36 ± 0.26^e^	121.62 ± 4.5^b^
Chicken	0.033 ± 0.001^e^	0.035 ± 0.004^c^	3.58 ± 0.30^a^	7.05 ± 1.06^f^	21.53 ± 0.27^g^
Turkey meat	0.039 ± 0.004^d^	0.039 ± 0.002^c^	3.98 ± 0.52^a^	11.96 ± 2.23^e^	47.71 ± 1.23^d^
Fish	4.28 ± 0.13^b^	0.042 ± 0.003^b^	0.042 ± 0.002^c^	8.57 ± 0.13^f^	21.44 ± 0.42^g^
Shrimp	0.042 ± 0.001^d^	0.042 ± 0.002^b^	4.07 ± 1.23^a^	50.59 ± 4.22^a^	59.02 ± 1.25^c^
Eggplant	0.043 ± 0.001^d^	0.043 ± 0.001^b^	0.043 ± 0.001^c^	39.09 ± 0.15^b^	21.72 ± 4.34^g^
Green vegetables	0.042 ± 0.001^d^	0.042 ± 0.002^b^	0.042 ± 0.002^c^	8.42 ± 0.41^f^	21.26 ± 0.42^g^
Tomato paste	4.03 ± 0.26^b^	0.41 ± 0.01^a^	4.17 ± 1.30^a^	20.85 ± 1.24^c^	16.70 ± 0.27^h^
Spice	0.043 ± 0.001^d^	0.043 ± 0.002^b^	0.043 ± 0.001^c^	8.11 ± 0.44^f^	12.93 ± 0.51^j^
Oil	0.005 ± 0.001^f^	0.005 ± 0.001^e^	1.0 ± 0.39^b^	1.0 ± 0.05^g^	0.98 ± 0.03^l^

*Note:* Wave length: As: 228.812; Cd: 214.438; Pb: 261.418; Cu: 324.754; Zn: 213.856 nm. Different letters in each column show significant differences at the level of *p* < 0.05.

Rice is regarded as a principal agricultural product and a pervasive foodstuff in Asian countries, including Iran (Zhuang et al. 2009). In a study conducted in the Dabaoshan mining area of South China, Pb and Cd in cultivated rice were approximately 8 and 6.5 times higher than the standard limit, respectively. This finding suggests that rice possesses a notable capacity to absorb Pb and Cd from contaminated soil (Zhuang et al. 2009). The variation in heavy metals of raw rice can be attributed to the type of rice, the method of irrigation, the type and pH of the soil, or the use of fertilizers and pesticides (Mao et al. 2019). The concentration of As, Cd, and Pb was found to be below the permissible limits recommended by the Joint FAO/WHO Committee (0.2, 0.4 and 0.2 mg/kg, respectively). In general, the concentration of metals in this study was found to be lower than the standard limits of FAO/WHO.

Heavy metals can enter animal feed and subsequently the animal bodies through contaminated food and water. Heavy metals are not biodegradable and, due to the nature of their accumulation, may become concentrated in various tissues (Abd‐Elghany et al. 2020). In the present study, the pattern of Pb in the examined meat samples was as follows: lamb meat > shrimp > turkey > chicken > fish = veal. The results of the present study indicate that the amount of Cu in meat samples was highest in shrimp, followed by veal, lamb meat, turkey, fish, and chicken. Additionally, the Cd pattern in meat samples was observed to be chicken < turkey < veal = fish = shrimp < lamb meat. It was estimated that the amount of Cd in all samples was less than the maximum permitted level of 5 mg/kg (Korish and Attia 2020). Kosimovna et al. (2022) revealed that the concentration of Pb, As, Cd, and Hg in veal meat was below the limit of detection, whereas the amount of Cu and Zn was in line with the standard range. The results of the analysis of Se, Pb, Cd, As, Co, Zn, Ni, Cu, and Cr in meat samples from sheep, veal, turkey, and ostrich in Iran indicate a notable difference in the concentration of selenium (Se), Nickel (Ni), cobalt (Dusengemungu et al. 2022), and chromium (Cr). Although Co concentration was found to exceed the maximum permissible limit (Raeeszadeh et al. 2022). The variation in contamination of meat and its products with heavy metals is due to the age of the animal (Njoga et al. 2021), the season of slaughter, the geographical location (Njoga et al. 2021), the contamination of pastures and water sources, and plants with toxic metals by overuse of organic fertilizers, and the use of acaricides for the treatment of external parasites (Zewdu Seyoum et al. 2016).

Edible oils are extracted from a variety of plant seeds, including corn, sesame, sunflower seeds, nuts, and others. They are widely used in cooking and in salads (Alrajhi and Idriss 2020). The joint FAO/WHO committee has announced the maximum acceptable levels for Cu, Zn, As, Cd, and Pb in edible oils as 30 and 60 mg/L and 0.1, 0.05, and 0.1 μg/kg, respectively (Famurewa et al. 2023). The amounts of heavy metals in oil samples have been attributed to various factors, including plant genetic characteristics, human activities, differences in soil contents, soil pollution, cultivation conditions, the method of oil extraction from raw materials, and oil refining (Famurewa et al. 2023; Shariatifar et al. 2022).

According to the FAO/WHO committee, the maximum permissible concentration of Pb in plant material is 10 mg/kg. According to the standards of China and the European Union, the maximum permissible concentration of Pb in spices is 3 and 0.1 mg/kg, respectively (Shim et al. 2019). In the present study, heavy metals in the spices were less than the established standard limit. the variation in metal concentration of spices was attributed to the variation in soil types, agricultural practices (the type of cultivation and irrigation; Tefera and Teklewold 2021), the specific plant compounds utilized in the preparation of spices, and geographical conditions (Bala et al. 2024).

The As, Cd, Pb, Cu, and Zn in tomato paste were 4.03, 0.41, 4.17, 20.85, and 16.70 μg/kg. In the study of Izah and Aigberua (2020), the concentration of Cr, Cu, Mn, and Zn in tomatoes was estimated to be between 0.396 and 0.896 mg/kg, 0.602 and 2.43 mg/kg, 1.77 and less than 0.001 mg/kg, and 2.048 and 0.11 mg/kg, respectively. In the study of Abbasi et al. (2020), the concentrations of Cd, Cr, and Co in tomato paste were determined to be within the range of 0.071–10.76, 0.037–13.32, and 0.004–0.839 mg/kg, respectively. The commercial brand of paste utilized, the type of raw materials employed, the methodology of transfer, storage, packaging, pH, and oxygen concentration may influence the quality of the coatings utilized, thereby potentially facilitating the release of metals from the packaging cans (Abbasi et al. 2020).

The permissible limits for Pb, Cd, and As set by CODEX in fruits and vegetables are 0.1, 0.05, and 0.1 mg/kg, respectively. However, the European Union standard sets the values for Pb and As at 0.2 mg/kg (Caicedo‐Rivas et al. 2022). In the current study, the concentrations of Pb, Cd, and As in eggplant and green vegetables were below the permissible level. Luo et al. estimated concentrations of Pb, Cd, Cu, and Zn in eggplant, tomato, and pepper as 0.082, 0.021, 0.996, and 0.590 mg/kg, respectively, of which in 5.8% of the vegetables, Pb was higher than the permissible limit of the Chinese standard (Luo et al. 2017). The accumulation of metals in plants is due to human activities, such as mining and industrial processes, the entry of wastewater from industrial factories into agricultural lands, and the use of chemical fertilizers. The absorption of metals in the soil by plants is dependent on the type of plant, the type of soil, pH, humidity, the amount of micronutrients, and the time of harvest (Zwolak et al. 2019).

### 3.2 Heavy Metals in Cooked Foods

The presence of heavy metals during food processing and storage as well as the type of food packaging materials can be a significant factor in the contamination of food (El Bushuty and Shanshan 2018). The results showed that the concentration of As in cooked food was between 0.028 and 5.55 μg/kg dry weight for cooked rice (polo) and chicken barbecue, respectively (Table 2). The process of frying, steaming, and soaking could reduce the levels of As (Cheyns et al. 2017). In the present study, the As levels of fish, cooked meat, minced kebab, rice + lentil + meat, eggplant stew, and Qormeh‐sabzi stew were reduced compared with their original raw materials. The results indicate that the As concentration is lower than the standard limit of FAO/WHO. Wang et al. reported the concentrations of As, Cu, Zn, Cd, and Pb in meat samples to be 0.2183, 1.0747, 46.3887, 0.0000, and 0.0780 mg/kg, respectively (Wang et al. 2023). The pattern of mean concentration of Pb in cooked food samples was observed in the form of Gheimeh stew > fried fish = fried shrimp = minced kebab = Qormeh‐sabzi stew > bread > lamb meat = chicken > eggplant stew > Istambuli polo > rice + Gheimeh stew (lentil + meat) > cooked rice. Cu concentrations in samples ranged from 2.98 to 35.12 mg/kg, in rice + lentil + meat and turkey, respectively. The Zn ranged from 9.01 to 198.7 mg/kg in fried fish and minced kebab, respectively. In the present study, the concentration of Cd in consumed cooked foods was lower than that reported by Chowdhury and Alam (2023) and higher than that reported by Wang et al. (2023). The concentration of Pb in minced kebab, fried fish, and Gheimeh stew has increased in comparison with the concentration of Pb in their raw materials. This increase may be attributed to the addition of other ingredients during the preparation process. Madani et al. (2023) reported Pb in foods as 0.05 and 0.79 mg/kg, which exceeded the permitted levels of FAO/WHO. It has been demonstrated that the processing and cooking of foodstuffs can result in a reduction or increase in the concentration of heavy metals. It is possible that metal utensils and equipment used for grilling may contaminate food with heavy metals (Inobeme et al. 2020). Also, various cooking methods, including steaming, frying, grilling, and boiling, have been demonstrated to reduce the concentration of heavy metals in fish (Ulaganathan et al. 2022). It can be generally stated that the increase of each of the metals As, Cd, Pb, Cu, and Zn in cooked food compared with its raw materials can be attributed to the presence of those metals in the cooking environment, the equipment used, and different cooking methods (Diyarov et al. 2022). In the study of Okon et al. (2023), the elevated concentration of certain heavy metals in bread was attributed to seasonings utilized in bread production. The present study revealed a significant reduction in the levels of these metals in the cooked rice, which can be attributed to the cooking method (Jafari‐Moghadam et al. 2015).

**TABLE 2:** Concentration of heavy metals in cooked foods based on dry weight (mean ± SD).

Food	As (μg/kg)	Cd (μg/kg)	Pb (μg/kg)	Cu (mg/kg)	Zn (mg/kg)
Cooked rice	0.028 ± 0.001^e^	0.028 ± 0.001^d^	0.028 ± 0.001^f^	11.29 ± 0.5^e^	22.58 ± 2.82^f^
Lentil, rice with meat	0.029 ± 0.002^e^	0.029 ± 0.002^d^	0.029 ± 0.001^f^	2.98 ± 1.21^h^	23.86 ± 2.1^f^
Istanbuli polo	3.49 ± 0.48^b^	0.035 ± 0.002^c^	0.035 ± 0.005^e^	3.49 ± 0.3^h^	20.99 ± 2.37^f^
Lamb meat	0.044 ± 0.001^c^	0.044 ± 0.001^a^	0.044 ± 0.001^d^	8.52 ± 0.05^f^	98.74 ± 4.48^b^
Chicken	0.045 ± 0.001^c^	0.045 ± 0.001^a^	0.045 ± 0.001^d^	8.99 ± 0.26^f^	68.61 ± 4.57^c^
Turkey	0.044 ± 0.001^c^	0.044 ± 0.001^a^	3.91 ± 0.02^c^	35.12 ± 0.25^a^	35.27 ± 0.12^e^
Fried fish	0.045 ± 0.001^c^	0.045 ± 0.001^a^	4.58 ± 0.3^b^	13.90 ± 0.26^d^	9.01 ± 0.26^h^
Fried shrimp	0.042 ± 0.001^d^	0.042 ± 0.001^b^	4.26 ± 0.4^b^	12.80 ± 0.42^d^	34.15 ± 2.54^e^
Chicken barbecue	5.55 ± 2.64^a^	0.042 ± 0.001^b^	0.042 ± 0.003^d^	17.10 ± 4.27^b^	21.38 ± 4.27^f^
Minced kebab	0.040 ± 0.001^d^	0.040 ± 0.001^b^	4.05 ± 0.55^b^	7.97 ± 0.23^g^	198.70 ± 4.05^a^
Gheimeh stew	4.03 ± 0.52^c^	0.035 ± 0.001^c^	10.55 ± 0.35^a^	18.76 ± 2.03^b^	95.52 ± 3.51^b^
Eggplant stew	0.039 ± 0.003^d^	0.039 ± 0.002^b^	0.039 ± 0.001^e^	19.53 ± 0.63^b^	62.25 ± 6.21^c^
Qorme‐Sabzi stew	0.040 ± 0.003^d^	0.040 ± 0.001^b^	4.04 ± 0.52^b^	16.03 ± 0.23^c^	48.50 ± 1.98^d^
Bread	4.59 ± 0.78^a^	0.038 ± 0.003^b^	3.79 ± 0.12^c^	15.19 ± 0.79^c^	15.19 ± 0.57^g^

*Note:* Wave length: As: 228.812; Cd: 214.438; Pb: 261.418; Cu: 324.754; Zn: 213.856 nm. Different letters in each column show significant differences at the level of *p* < 0.05.

### 3.3 Heavy Metals in Beverages

The consumption of nonalcoholic beverages, including carbonated drinks and juice, has increased on a global scale in recent years (Shariatifar et al. 2022). As indicated by the WHO, the acceptable levels of Cu, Pb, and Cd in consumed beverages are 2, 0.01, and 0.001 mg/kg, respectively. In the current study, all heavy metals in beverages were lower than the standard limit (Table 3). Abdel‐Rahman et al. indicated that the levels of Pb and Cd in soft drinks and juices were below the detection limit, whereas the concentration of Cu ranged from 0.16 ± 0.01 to 0.56 ± 0.05 mg/kg. The low levels of these contaminants are likely the result of careful selection of raw materials and appropriate processing methods, including the use of suitable can packaging and well‐maintained production facilities (Abdel‐Rahman et al. 2019; Sani et al. 2023). In the study of Bamuwamye et al. (2022), the concentrations of As, Cd, Pb, and Cu in water were 0.011, undetectable, 0.178, and 0.019 mg/L, respectively. These researchers identified the pipes and facilities used for drinking water as the sources of Pb and nickel metals in drinking water. They identified mining operations and improper disposal of waste.

**TABLE 3:** Concentration of heavy metals in beverages (mean ± SD).

Beverages	As (μg/L)	Cd (μg/L)	Pb (μg/L)	Cu (mg/L)	Zn (mg/L)
Nonalcoholic beer	0.88 ± 0.20^a^	0.005 ± 0.001^b^	0.5 ± 0.09^b^	0.5 ± 0.01^a^	0.005 ± 0.001^c^
Doogh	0.5 ± 0.01^b^	0.005 ± 0.001^b^	1.00 ± 0.12^a^	0.005 ± 0.001^c^	2.16 ± 0.69^a^
Fruit juice	0.005 ± 0.001^d^	0.005 ± 0.001^b^	1.00 ± 0.21^a^	0.005 ± 0.001^c^	0.005 ± 0.001^c^
Soft drinks	0.005 ± 0.001^d^	0.005 ± 0.001^b^	0. 5 ± 0.01^b^	0.5 ± 0.01^a^	0.005 ± 0.001^c^
Water	0.01 ± 0.001^c^	0.001 ± 0.001^a^	0.001 ± 0.00^c^	0.001 ± 0.00^b^	0.04 ± 0.001^b^

*Note:* Wave length: As: 228.812; Cd: 214.438; Pb: 261.418; Cu: 324.754; Zn: 213.856 nm. Different letters in each column show significant differences at the level of *p* < 0.05.

### 3.4 Estimated Daily Intake, Hazard Quotient (HQ) and Hazard Index (HI) of Exposure to Heavy Metals in Cooked Foods

It is beneficial to assess the intake of heavy metals by estimating the amount consumed and the tolerable daily intake, as well as calculating the daily HQ and HI values recommended by international organizations (Abd‐Elghany et al. 2020). The HQ and HI of heavy metals were estimated to be less than one for all cooked foods (Table 4) which is in agreement with results of Jin et al. (2023), and Kharazi et al. (2021). Therefore, the ingestion of foods will not have any deleterious effects on the consumer. The HI and HQ results of the current study is not compatible with results of Yi et al. (2017) (food web), and Kazemi et al. (2022) (edible tissue of marine fish). Han et al. (2022) determined the HQ values for As, Cd, Cr, Cu, Hg, Ni, and Pb in poultry meat to be 0.011, 0.005, 0.000, 0.038, 0.051, and 0.005, respectively. In the study of Abd‐Elghany et al., the amount of HQ for Hg, Cd, and Cr in red meat was 1.74, 0.64, 0.17, and 0.066, respectively, and the value of HI for lamb meat samples was estimated as 2.79 (Abd‐Elghany et al. 2020). In Njoga et al. (2021)'s research, the amount of HQ for As, Cd, and Pb was estimated as 0.091, 0.009, and 0.123, respectively.

**TABLE 4:** Estimated daily intake, hazard quotient (HQ) and hazard index (HI) of exposure to heavy metals in cooked foods.

Foodstuff	Parameters	As	Cd	Pb	Cu	Zn
Cooked rice	EDI	7.19 × 10^−8^	7.19 × 10^−8^	7.19 × 10^−8^	2.90 × 10^−5^	5.80 × 10^−5^
	HQ	2.39 × 10^−4^	7.19 × 10^−5^	2.05 × 10^−5^	7.25 × 10^−5^	1.39 × 10^−4^
	HI = ƩHQ	5.98 × 10^−4^				
Minced kebab	EDI	5.68 × 10^−7^	5.68 × 10^−9^	5.76 × 10^−7^	1.13 × 10^−6^	2.82 × 10^−5^
	HQ	1.89 × 10^−5^	5.68 × 10^−6^	1.64 × 10^−4^	2.83 × 10^−6^	9.41 × 10^−5^
	HI = ƩHQ	2.86 × 10^−4^				
Adas polo with meat	EDI	5.15 × 10^−9^	5.15 × 10^−9^	5.15 × 10^−9^	5.29 × 10^−7^	4.24 × 10^−6^
	HQ	1.71 × 10^−5^	5.15 × 10^−6^	1.47 × 10^−6^	1.32 × 10^−6^	1.41 × 10^−5^
	HI = ƩHQ	3.92 × 10^−5^				
Istanbuli polo	EDI	2.48 × 10^−6^	2.48 × 10^−8^	2.48 × 10^−8^	2.48 × 10^−6^	1.49 × 10^−5^
	HQ	8.27 × 10^−3^	2.48 × 10^−5^	7.11 × 10^−6^	6.20 × 10^−6^	4.97 × 10^−5^
	HI = ƩHQ	8.36 × 10^−3^				
Gheimeh stew	EDI	5.37 × 10^−7^	4.66 × 10^−9^	1.40 × 10^−6^	2.50 × 10^−6^	1.27 × 10^−5^
	HQ	1.79 × 10^−3^	1.79 × 10^−3^	4.01 × 10^−4^	6.25 × 10^−6^	4.24 × 10^−5^
	HI = ƩHQ	2.24 × 10^−3^				
Eggplant stew	EDI	5.20 × 10^−9^	5.20 × 10^−9^	5.20 × 10^−9^	2.60 × 10^−6^	8.33 × 10^−6^
	HQ	1.73 × 10^−5^	5.20 × 10^−6^	1.48 × 10^−6^	6.51 × 10^−6^	2.77 × 10^−5^
	HI = ƩHQ	5.83 × 10^−5^				
Qorme‐Sabzi stew	EDI	5.33 × 10^−9^	5.33 × 10^−9^	5.38 × 10^−7^	2.13 × 10^−6^	4.46 × 10^−6^
	HQ	1.77 × 10^−5^	5.33 × 10^−6^	1.53 × 10^−4^	5.34 × 10^−6^	2.15 × 10^−5^
	HI = ƩHQ	2.03 × 10^−4^				
Chicken barbecue	EDI	9.62 × 10^−7^	7.28 × 10^−9^	7.28 × 10^−9^	2.96 × 10^−6^	3.70 × 10^−6^
	HQ	3.20 × 10^−3^	7.28 × 10^−6^	2.08 × 10^−6^	7.41 × 10^−6^	1.32 × 10^−5^
	HI = ƩHQ	2.23 × 10^−3^				
Chicken	EDI	1.52 × 10^−8^	1.52 × 10^−8^	1.52 × 10^−8^	3.03 × 10^−6^	2.31 × 10^−5^
	HQ	5.06 × 10^−5^	1.52 × 10^−5^	4.34 × 10^−6^	7.59 × 10^−6^	7.72 × 10^−5^
	HI = ƩHQ	1.55 × 10^−4^				
Fried fish	EDI	1.20 × 10^−8^	1.20 × 10^−8^	1.22 × 10^−6^	3.70 × 10^−6^	2.40 × 10^−6^
	HQ	4.00 × 10^−5^	1.20 × 10^−5^	3.48 × 10^−4^	9.26 × 10^−6^	8.00 × 10^−6^
	HI = ƩHQ	4.18 × 10^−4^				
Lamb meat	EDI	1.95 × 10^−9^	1.95 × 10^−9^	1.95 × 10^−9^	3.78 × 10^−7^	4.38 × 10^−6^
	HQ	6.51 × 10^−6^	1.95 × 10^−6^	5.58 × 10^−7^	9.46 × 10^−7^	1.46 × 10^−5^
	HI = ƩHQ	2.46 × 10^−5^				
Fried shrimp	EDI	1.30 × 10^−9^	1.30 × 10^−9^	1.32 × 10^−7^	3.98 × 10^−7^	1.06 × 10^−6^
	HQ	4.35 × 10^−6^	1.30 × 10^−6^	3.78 × 10^−5^	9.95 × 10^−7^	3.54 × 10^−6^
	HI = ƩHQ	4.80 × 10^−5^				
Turkey	EDI	2.93 × 10^−9^	2.93 × 10^−9^	2.60 × 10^−7^	2.34 × 10^−6^	2.35 × 10^−6^
	HQ	9.77 × 10^−6^	2.93 × 10^−6^	7.44 × 10^−5^	5.85 × 10^−6^	7.83 × 10^−6^
	HI = ƩHQ	1.00 × 10^−4^				

### 3.5 Estimated Daily Intake, Hazard Quotient (HQ) and Hazard Index (HI) of Exposure to Heavy Metals in Beverages

The HQ and HI of beverages consumed in the present study were found to be less than one (Table 5), indicating that there is no risk to the health of the consumers of these beverages, which is compatible with the results of Taiwo et al. (2020) (drinks), Yüksel et al. (2023) (canned nonalcoholic drink), and Shariatifar et al. (2022) (commercial soft drinks). In the study of Seleem et al. (2021), the HQ for Pb was 1.93, for Cd 0.39, for Cu and Zn 0.00, and for As 1.83 in drinking water. The HI for metals, including Fe, Mn, Pb, Cd, Ni, Cu, Zn, Cr, and As, was determined to be 5.63 for drinking water, a value that is considerably higher than that observed in the present study. In the study of drinking water in Uganda by Bamuwamye et al. (2022), the hazard quotient (HQ) value for Pb metal was 14.127, for As 1.048, and for Cd, Cu, and Zn elements less than 1. The hazard index (HI) value for all heavy metals tested was 15.0488, which is much higher compared with the values measured by the present study. In the study conducted by Kowalska et al. (2020), the HQ for As, Cd, and Pb metals in all juices was found to be less than one, with a range of 0.1158–0.1430, 0.1086–0.1194, and 0.0022–0.0027, respectively.

**TABLE 5:** Estimated daily intake, hazard quotient (HQ) and hazard index (HI) of exposure to heavy metals in beverages.

Beverages	Parameters	As	Cd	Pb	Cu	Zn
Nonalcoholic beer	EDI	1.89 × 10^−8^	1.07 × 10^−10^	1.07 × 10^−10^	1.07 × 10^−8^	1.07 × 10^−10^
	HQ	6.03 × 10^−5^	1.07 × 10^−7^	3.07 × 10^−6^	2.68 × 10^−8^	3.58 × 10^−10^
	HI = ƩHQ	6.63 × 10^−5^				
Doogh	EDI	1.07 × 10^−8^	1.07 × 10^−10^	2.15 × 10^−8^	1.07 × 10^−10^	4.64 × 10^−8^
	HQ	3.58 × 10^−5^	1.07 × 10^−7^	6.14 × 10^−6^	2.68 × 10^−10^	1.54 × 10^−7^
	HI = ƩHQ	4.22 × 10^−5^				
Fruit juice	EDI	7.82 × 10^−11^	7.82 × 10^−11^	1.56 × 10^−8^	7.82 × 10^−11^	7.82 × 10^−11^
	HQ	2.60 × 10^−7^	7.82 × 10^−8^	4.46 × 10^−6^	1.95 × 10^−10^	2.60 × 10^−10^
	HI = ƩHQ	4.80 × 10^−6^				
Soft drinks	EDI	1.07 × 10^−10^	1.07 × 10^−10^	1.07 × 10^−8^	1.07 × 10^−8^	1.07 × 10^−10^
	HQ	3.58 × 10^−7^	1.07 × 10^−7^	3.07 × 10^−6^	2.68 × 10^−8^	3.58 × 10^−10^
	HI = ƩHQ	3.56 × 10^−6^				
Water	EDI	3.25 × 10^−10^	3.25 × 10^−11^	3.25 × 10^−11^	3.25 × 10^−11^	1.30 × 10^−9^
	HQ	1.08 × 10^−6^	3.25 × 10^−8^	9.30 × 10^−9^	8.14 × 10^−11^	4.34 × 10^−9^
	HI = ƩHQ	1.13 × 10^−6^				

## 4 Conclusion

Heavy metal exposure among miners poses significant health risks, including both noncarcinogenic and carcinogenic effects. The implementation of effective risk assessment and mitigation strategies is imperative to ensure the protection of miners and surrounding communities. The findings of this study indicate that the concentrations of As, Cd, Pb, Cu, and Zn in all raw and cooked foods consumed by employees were below the maximum permissible limits established by international regulatory bodies, including the FAO/WHO. The presence of trace amounts of these heavy metals in food products is likely attributable to contamination occurring at various stages of the food production and processing chain, from farm to table. Additionally, the calculated hazard quotient (HQ) and hazard index (HI) values suggest that the consumption of cooked food and beverages by employees does not pose a significant health risk. However, regular monitoring of food products is essential to ensure the continued assessment of heavy metal contamination and to mitigate potential long‐term health risks. Further research is necessary to address existing gaps in understanding the long‐term health impacts of heavy metal exposure and to develop more effective remediation techniques.

## Author Contributions

All authors contributed to the study's conception and design. Material preparation, data collection, and analysis were performed by Maryam Rostamzadeh, Elham Khalili Sadrabad, Fateme Akrami Mohajeri, Arefeh Dehghani‐tafti and Elaheh Askari. The first draft of the manuscript was written by Maryam Rostamzadeh, Elaheh Askari and Elham Khalili Sadrabad. All authors commented on previous versions of the manuscript. All authors read and approved the final manuscript.

## Ethics Statement

The authors have nothing to report. Ethics Approval: IR.SSU.REC.1402.033.

## Conflicts of Interest

The authors declare no conflicts of interest.

## Data Availability

The data that support the findings of this study are available on request from the corresponding author. The data are not publicly available due to privacy or ethical restrictions.
